# *Schistosoma mansoni* and soil-transmitted helminths among schoolchildren in An-Nadirah District, Ibb Governorate, Yemen after a decade of preventive chemotherapy

**DOI:** 10.1371/journal.pone.0273503

**Published:** 2022-08-25

**Authors:** Walid M. S. Al-Murisi, Abdulsalam M. Al-Mekhlafi, Mohammed A. K. Mahdy, Sami Ahmed Al-Haidari, Dhekra A. Annuzaili, Ahmed Ali Qaid Thabit

**Affiliations:** 1 Department of Parasitology, Faculty of Medicine, Sana’a University, Sana’a, Yemen; 2 Tropical Disease Research Center, Faculty of Medicine, University of Science and Technology, Sana’a, Yemen; 3 Diseases Control & Surveillance, Ministry of Public Health and Population, Sana’a, Yemen; 4 Primary Health Care Department, Ministry of Public Health and Population, Aden, Yemen; 5 Communicable Diseases Department, World Health Organization, Sana’a, Yemen; Dokkyo Medical University, JAPAN

## Abstract

The Ministry of Public Health in Yemen continues the implementation of school and community–based preventive chemotherapy with praziquantel and albendazole for the control and elimination of schistosomiasis and soil-transmitted helminths (STH). The latest remapping to update the distribution of schistosomiasis and STH was conducted seven years ago. This study aimed to estimate the prevalence, intensity and associated risk factors of *Schistosoma mansoni* and STH among schoolchildren in An-Nadirah District, Ibb Governorate, Yemen. A cross-sectional study was carried out among schoolchildren aged 6–15 years in four selected schools. Biological, demographic, socioeconomic and environmental data were collected using a pre-tested questionnaire. *S*. *mansoni* and STH eggs were detected and counted by the microscopic examination of Kato-Katz fecal smears. Out of 417 schoolchildren, 17.0% were infected with at least one intestinal helminth. Prevalence of *S*. *mansoni* and STH were 6.5% and 9.1%, respectively. The most prevalent parasite among STH was *Ascaris lumbricoides* (8.4%). Unemployed fathers (Adjusted Odds Ratio (AOR) = 3.2; 95% Confidence interval (CI): 1.23, 8.52; *P* = 0.018), eating exposed food (AOR: 2.9; 95%CI = 1.24, 6.89; *P* = 0.014), not washing hands before eating and after defecation (AOR: 4.8; 95%CI = 1.77, 12.81; *P* = 0.002), and schools located close to water stream (AOR: 22.1; 95%CI = 5.12, 95.46; *P* <0.001) were independent risk factors of ascariasis. Swimming in ponds/stream (AOR: 3.9; 95%CI = 1.63, 9.55; *P* = 0.002), and schools close to the stream (AOR: 24.7; 95%CI = 3.05, 200.07; *P* = 0.003) were independent risk factors of intestinal schistosomiasis. The present study does not indicate a reduction in the prevalence of intestinal schistosomiasis in this rural area since the latest remapping conducted in 2014, although ascariasis was reduced by half. The prevalence of the two parasites was highly focal in areas close to the valley, suggesting a significant role of the stream in sustaining and accelerating the parasitic infection. Children practicing swimming and having poor hygienic practices were at high exposure to *S*. *mansoni* and *A*. *lumbricoides*, respectively. Water, Sanitation and Hygiene intervention, school–based health education, and snail control, in addition to mass drug administration, will help in the interruption of transmission of schistosomiasis and STH.

## 1. Introduction

*Schistosoma mansoni* and soil-transmitted helminths (STH) (*Ascaris lumbricoides*, *Trichuris trichiura*, and hookworms) are neglected tropical diseases (NTDs) causing morbidity and mortality worldwide. The 2015 global estimates showed that schistosomiasis, ascariasis, trichuriasis and hookworm infections affect 0.25 billion (95% uncertainty interval (95%UI): 0.21–0.32 billion), 0.76 billion (95%UI: 0.68–0.86 billion), 0.46 billion (95%UI: 0.43–0.50 billion) and 0.43 billion (0.40–0.47 billion), respectively [1]. These parasitic infections have adverse effect on the nutritional status, cognitive function, and physical and mental development of children [2–4].

*S*. *mansoni* and STH have a widespread prevalence in communities with poor sanitary condition, low-income, and overcrowded places [5]. Furthermore, unsafe drinking and irrigation water sources are risk factors for *S*. *mansoni* [6–9]. Preventive chemotherapy through mass drug administration (MDA) is the main intervention for the control and elimination of schistosomiasis and STH [10–13]. Ministry of Public Health, Yemen, has been implementing nationwide MDA, with praziquantel for schistosomiasis and albendazole for STH through school and community channels since 2010 [14]. The nationwide remapping survey, conducted in 2014, showed that overall prevalence of schistosomiasis and STH was reduced to 3.2% and 8.8%, respectively [15]. However, there is no updated picture about *S*. *mansoni* and STH in the last seven years. It is noteworthy that Yemen is suffering from ongoing war started in early 2015, which might affect the distribution of *S*. *mansoni* and STH. Besides, foci with high prevalence of *S*. *mansoni* and STH have been reported [16, 17]. The present study aimed to determine the prevalence of STH and *S*. *mansoni*, and identify their risk factors among schoolchildren in An-Nadirah District, an endemic area in Ibb Governorate, Yemen.

## 2. Materials and methods

### 2.1. Study area

An-Nadirah District is a mountainous area in Ibb Governorate, Yemen. It is located at an altitude of 2153 meters above sea level along Bana Valley, which combines a permanent stream flowing toward Abyan Governorate. Stream, underground wells and rains are the main water sources for domestic and irrigation purposes. The district is endemic for *S*. *mansoni* and STH and has been targeted by the preventive chemotherapy. Therefore, it was purposively selected as an example to assess the impact of the MDA campaigns on the prevalence of *S*. *mansoni* and STH.

### 2.2. Study design and population

A cross-sectional study was carried out among schoolchildren aged 6–15 years. The study targeted four primary schools with a total of 1429 students attending school during the scholarly year 2019.

### 2.3. Sample size and sampling strategy

The minimum sample size was calculated EpiInfo programme (https://www.cdc.gov/epiinfo/index.html) using the following parameters; 95% Confidence interval and precision of ± 5%. The highest prevalence reported by the latest remapping, conducted in 2014, was 14% for *A*. *lumbricoides* [15], which was used for the sample size calculation. The sample size was inflated by the design effect of two and an expected non-response rate of 10% due to absenteeism or other reasons. The total sample size required was 408 schoolchildren, although 417 children were enrolled from four schools that were selected using cluster sampling approach. The students were first ranked according to their class levels (grade 1–9). A proportional sample was then selected from each class by systemic random sampling using school records as the sampling frame.

### 2.4. Questionnaire

A pre-tested questionnaire was used to collect information on the demographic, socio-economic background, hygienic practices and environment through face-to-face interview with schoolchildren. Information about personal hygiene, fingernails trimming and walking barefoot was collected by direct observation.

### 2.5. Parasitological examination

A single fecal specimen was obtained from each study participant and two Kato-Katz thick smears were prepared from each fecal sample in the field following a standard protocol [18]. The smears were examined microscopically for the presence of hookworm’s eggs in a half to one hour after the preparation. Then, Kato-Katz thick smears were transferred to the Department of Parasitology, Faculty of Medicine, Sana’a University, and examined for STH and *S*. *mansoni* eggs using light microscopy by two experienced laboratory technologists, independently. Smears with discrepancies in results between the two readers were re-examined by a third lab technician who was blinded to the first results. Following the WHO guidelines, the intensity of *S*. *mansoni* was classified into light (1–99 eggs/ gram of stool(epg)), moderate (100–399 epg) and high (≥ 400 epg), while the intensity of *A*. *lumbricoides* was classified into light (1–4,999 epg), moderate (5,000–49,999 epg), and high (≥ 50,000 epg) [19]. The area was classified according to the prevalence of *S*. *mansoni* into high–risk (prevalence ≥ 50%), moderate–risk (prevalence = 10–50%), and low–risk (prevalence = 1- <10%), while the area was classified to high and low risk if the prevalence of STH was ≥ 50% and ≥ 20 - < 50%, respectively [20].

### 2.6. Data analysis

Data analysis was conducted by using the IBM SPSS Statistics version 24 (IBM Corp., Armonk, NY, USA). Data were presented in frequency tables. Prevalence was reported with its corresponding 95% confidence interval (CI). The association between independent and dependent variables was tested using Chi-square or Fisher’s exact tests when applicable with reporting odds ratio (OR) and its corresponding 95% CI. Multivariable analysis using binary logistic regression model was developed for all variables included in the univariable analysis and adjusted odds ratio (AOR) with its corresponding 95% CI were reported. P-value < 0.05 was considered significant.

### 2.7. Ethical clearance

This research protocol was approved by the Research and Ethics Committee (REC) of the Faculty of Medicine and Health Sciences, Sana’a University. Permission was obtained from each schoolmaster/ schoolmistress after clarifying the importance of the study. Each child was voluntarily involved after explaining the nature of their participation in the study in a way that the child can understand and give his/her assent. Since the study protocol was conducted in schools where children’s parents/guardians were not existing, no informed consent was obtained, although those parents/guardians were informed about the study and had the right to refuse the participation of their children. The lack of child’s caretaker consent was approved by the REC. Anonymity, dignity and privacy of each child and his/her family were protected.

## 3. Results

### 3.1. Characteristics of subjects

A total of 417 schoolchildren aged 6–15 years with a mean age of 11.2 years have participated in this study (54.4% were males and 45.6% were females). Overall, more than three-quarters (83.7%) of fathers were educated with at least a primary school certificate while about two-thirds of mothers were uneducated. A total of 157 (37.6%) and 131 (31.4%) children were living in houses without access to piped water and improved sanitation, respectively (Table 1).

**Table 1:** Characteristics of schoolchildren (n = 417).

Characteristics	n (%)
**Gender**	
Male	227 (54.4)
Female	190 (45.6)
**Age groups (years)**	
≤ 10	157 (37.6)
> 10 (11–15)	260 (62.4)
**Fathers’ education level**	
Educated	349 (83.7)
Uneducated	68 (16.3)
**Mothers’ education level**	
Educated	143 (34.3)
Uneducated	273 (65.5)
**Fathers’ occupational status**	
Employed	213 (51.1)
Unemployed	204 (48.9)
**Family size**	
< 8 members	216 (51.8)
≥8 members	201 (48.2)
**Toilet in home**	
Available	389 (93.3)
Not available	28 (6.7)
**Toilet in school**	
Available	355 (85.1)
Not available	62 (14.9)
**Source of household’s water**	
Piped water	260 (62.4)
Other	157 (37.6)
**Sanitation** *	
Improved	286 (68.6)
Unimproved	131 (31.4)

* Improved sanitation (Flush/pour flush toilet to piped sewer system or Pit latrine) and unimproved sanitation (no toilet or Flush/pour flush toilet to open area).

### 3.2. Prevalence and distribution of Intestinal helminths

The prevalence of intestinal helminths among schoolchildren was 17%. For soil-transmitted helminths, the prevalence of *Ascaris lumbricoides*, *Trichuris trichiura*, and hookworm was 8.4%, 0.7%, and 0.5%, respectively. The prevalence of *Schistosoma mansoni* was 6.5%. The majority of infections with *S*. *mansoni* or *A*. *lumbricoides* were light infections (Table 2). Children from schools close to stream had higher infection rate compared to other schools (Table 3).

**Table 2:** Prevalence of intestinal helminths among schoolchildren in An-Nadirah District, Ibb Governorate, Yemen (n = 417).

Parasites	Positive	95%CI
	n(%)	(%)
Intestinal helminths’ infection	71(17.0)	(13.4, 20.6)
STH infection	38(9.1)	(6.2, 11.8)
**Type of infection**		
*A*. *lumbricoides*	35(8.4)	(5.3, 10.7)
*T*. *trichiura*	3(0.7)	(0.2, 2.0)
Hookworms	2(0.5)	(0.0, 1.0)
*S*. *mansoni*	27(6.5)	(3.6, 8.4)
*H*. *nana*	20(4.8)	(3.0, 7.1)
*E*. *vermicularis*	4(1)	(0.1, 2.0)
*Fasciola* spp.	2(0.5)	(0.0, 2.0)
**Mixed infection**		
*S*. *mansoni* & *A*. *lumbricoides*	9(2.2)	(0.6, 3.4)
*S*. *mansoni* & *T*. *trichiura*	2(0.5)	(0.0, 1.0)
**Intensity of *S*. *mansoni*** ^*^		
Light	21(5.0)	(11.4, 24.7)
Moderate	6(1.5)	
**Intensity of *A*. *lumbricoides*** ^***#***^		
Light	34(8.2)	(17.1, 32.9)
Moderate	1(0.2)	

*; eggs number in this study ranged from 24–96 eggs per gram of stool for light infection and 126–312 eggs per gram of stool for moderate infection

#; eggs number in this study ranged from 24–1272 eggs per gram of stool for light infection and only one case was moderate infection (37200 eggs per gram of stool).

**Table 3:** Prevalence of intestinal helminths among children in the selected schools according to their distance from the stream.

Name of school	Distance from the stream	N	Intestinal helminths	STH	*S*. *mansoni*	*A*. *lumbricoides*
			n(%)	n(%)	n(%)	n(%)
**Abdulrahman Bin Awf**	191 m	122	45(36.9)	26(21.3)	22(18.0)	25(20.5)
**Al-Fajer**	606 m	98	18(18.4)	9(9.2)	4(4.1)	7(7.1)
**Al-Eslah**	8.1 km	62	0(0.0)	0(0.0)	0(0.0)	0(0.0)
**Al-Shaheed Ayash**	11.6 km	135	8(5.9)	3(2.2)	1(0.7)	3(2.2)

### 3.3. Factors associated with *A*. *lumbricoides* infection

Univariable analysis identified a significant association between *A*. *lumbricoides* infection and uneducated father (OR = 3.0; 95%CI = 1.43, 6.45; P = 0.005), not washing hands before eating and after defecation (OR: 4.3; 95%CI = 2.04, 9.15; P <0.001), eating unwashed vegetables and fruits (OR: 2.9; 95%CI = 1.38, 6.01; P = 0.006), eating exposed food (OR: 4.6; 95%CI = 2.25, 9.41; P <0.001), and schools close to the stream (OR: 11.0; 95%CI = 3.31, 36.56; P<0.001). Multivariable analysis using binary logistic regression model identified unemployed father (AOR: 3.2; 95%CI = 1.23, 8.52; *P* = 0.018), not washing hands before eating and after defecation (AOR: 4.8; 95%CI = 1.77, 12.81; P = 0.002), eating exposed food (AOR: 2.9; 95%CI = 1.24, 6.89; P = 0.014), and schools close to the stream (AOR: 22.1; 95%CI = 5.12, 95.46; P <0.001) as independent risk factors of *A*. *lumbricoides* infection (Table 4).

**Table 4:** Factors associated with *A*. *lumbricoides* among schoolchildren in An-Nadirah District, Ibb Governorate, Yemen.

Variables	N	n(%)	OR(95%CI)	AOR(95%CI)	P value
**Gender**					
Male	227	15(6.6)	Reference		
Female	190	20(10.5)	0.6(0.30, 1.21)	0.9(0.39, 2.11)	0.831
**Age groups (years)**					
≤10	157	12(7.6)	Reference		
>10	260	23(8.8)	0.9(0.41, 1.76)	1.3(0.49, 3.30)	0.630
**Father’s education**					
Educated	349	23(6.6)	Reference		
Uneducated	68	12(17.6)	3.0(1.43, 6.45)	1.3(0.44, 3.70)	0.648
**Mother’s education**					
Educated	143	12(8.4)	Reference		
Uneducated	273	23(8.4)	1.0(0.48, 2.08)	0.4(0.17, 1.17)	0.102
**Father’s occupation**					
Employed	213	15(7.0)	Reference		
Unemployed	204	20(9.8)	1.4(0.71, 2.89)	3.2(1.23, 8.52)	0.018
**Family size**					
<8 members	216	20(9.3)	Reference		
≥8 members	201	15(7.5)	0.8(0.39, 1.59)	0.7(0.30, 1.71)	0.452
**Household’s water**					
Piped water	260	19(7.3)	Reference		
Other	157	16(10.2)	1.4(0.72, 2.89)	0.6(0.27, 1.58)	0.339
**Sanitation**					
Improved	286	26(9.1)	Reference		
Unimproved	131	9(6.9)	0.7(0.34, 1.62)	1.0(0.38, 2.54)	0.965
**Hand washing before eating and after defecation**					
Yes	358	22(6.1)	Reference		
No	59	13(22)	4.3(2.04, 9.15)	4.8(1.77, 12.81)	0.002
**Eating unwashed vegetables & fruits**					
No	339	22(6.5)	Reference		
Yes	78	13(16.7)	2.9(1.38, 6.01)	1.8(0.73, 4.55)	0.196
**Eating exposed food**					
No	335	18(5.4)	Reference		
Yes	82	17(20.7)	4.6(2.25, 9.41)	2.9(1.24, 6.89)	0.014
**Nails clipping**					
Yes	328	23(7.0)	Reference		
No	89	12(13.5)	2.1(0.99, 4.34)	1.0(0.38, 2.40)	0.926
**Shoes wearing**					
Yes	355	30(8.5)	Reference		
No	62	5(8.1)	1(0.35, 2.55)	0.8(0.25, 2.62)	0.715
**Swimming in ponds/stream**					
No	311	30(9.6)	Reference		
Yes	106	5(4.7)	0.5(0.18, 1.23)	0.5(0.15, 1.48)	0.196
**Location of schools**					
Close (191–606 m)	220	32(14.5)	Reference		
Far (> 8 km)	197	3(1.5)	11.0(3.31, 36.56)	22.1(5.12, 95.46)	<0.001

N; the number of samples examined, n; the number of positive samples, OR; Odds ratio, AOR; adjusted OR, CI; confidence interval, other sources of drinking water; stream, wells, dams…etc. *; Improved sanitation (Flush/pour flush toilet to piped sewer system or Pit latrine) and unimproved sanitation (no toilet or Flush/pour flush toilet to open area), #; How far is the school from stream.

### 3.4. Risk factors associated with *S*. *mansoni* infection

Univariable analysis identified an association between *S*. *mansoni* infection and uneducated father (OR: 3.4; 95%CI = 1.47, 7.71; P = 0.006), swimming or having contact with stream water (OR: 3.5; 95%CI = 1.58, 7.69; P = 0.002), and schools close to the stream (OR: 26.3; 95%CI = 3.53, 195.49; P <0.001). Multivariable analysis using binary logistic regression model identified swimming in ponds/stream (AOR: 3.9; 95%CI = 1.63, 9.55; P = 0.002), and schools close to the stream (AOR: 24.7; 95%CI = 3.05, 200.07; P = 0.003) as independent risk factors of *S*. *mansoni* infection (Table 5).

**Table 5:** Factors associated with *S*. *mansoni* among schoolchildren in An-Nadirah District, Ibb Governorate, Yemen.

Variable	N	n(%)	OR(95%CI)	AOR(95%CI)	P value
**Gender**					
Male	227	11(4.8)	Reference		
Female	190	16(8.4)	0.6(0.25, 1.22)	1.1(0.38, 3.00)	0.890
**Age groups (years)**					
≤10	157	8(5.1)	Reference		
>10	260	19(7.3)	0.7(0.29, 1.60)	1.1(0.38, 3.00)	0.897
**Father’s education**					
Educated	349	17(4.9)	Reference		
Uneducated	68	10(14.7)	3.4(1.47, 7.71)	2.5(0.78, 8.05)	0.125
**Mother’s education**					
Educated	143	9(6.3)	Reference		
Uneducated	273	18(6.6)	1.1(0.46, 2.40)	0.5(0.18, 1.47)	0.214
**Father’s occupation**					
Employed	213	11(5.2)	Reference		
Unemployed	204	14(6.8)	0.8(0.35, 1.77)	0.8(0.29, 2.33)	0.720
**Family size**					
<8 members	216	16(7.4)	Reference		
≥8 members	201	11(5.5)	0.7(0.33, 1.59)	0.8(0.31, 1.99)	0.605
**Household’s water**					
Piped water	260	14(5.4)	Reference		
Other	157	13(8.3)	1.6(0.73, 3.45)	1.5(0.60, 3.62)	0.394
**Sanitation** *					
Improved	286	23(8.0)	Reference		
Unimproved	131	4(3.1)	0.4(0.12, 1.06)	0.5(0.15, 1.63)	0.246
**Swimming in ponds/stream**					
No	311	13(4.2)	Reference		
Yes	106	14(13.2)	3.5(1.58, 7.69)	4.1(1.79, 10.28)	0.001
**Location of schools** ^**#**^					
Close (191–606 m)	220	26(11.8)	Reference		
Far (> 8 km)	197	1(0.5)	26.3(3.53, 195.49)	22.6(2.80, 183.09)	0.003

**N**; the number of samples examined, n; the number of positive samples, OR; Odds ratio, AOR; adjusted OR, CI; confidence interval, other sources of drinking water; stream, wells, dams…etc.

*; Improved sanitation (Flush/pour flush toilet to piped sewer system or Pit latrine) and unimproved sanitation (no toilet or Flush/pour flush toilet to open area)

#; How far is the school from stream.

## 4. Discussion

The present study aimed to determine the prevalence of STH and *S*. *mansoni* in an endemic area after a decade of regular school-based and community-based preventive chemotherapy. The prevalence of STH was 9.1% with *A*. *lumbricoides* being the most common STH (8.1%). This finding indicates reduction in the prevalence of STH among schoolchildren in An-Nadirah District compared to a previous survey conducted in the same area (8.1% *vs* 14%) [14], classifying this district as a low risk area (Low risk: <20%) [20]. This, in turn, indicates that MDA has achieved success in the control of STH. The predominance of *A*. *lumbricoides* compared to other STH is consistent with previous studies conducted in Yemen [14, 15], Middle East and north Africa region [21].

*A*. *lumbricoides* infection was higher among children from schools that are close to the stream than children from other schools. Besides, multivariable analysis identified schools close to the stream as independent risk factor of ascariasis. The statistical analysis does not suggest household’s drinking water or contact with the stream as predictors of ascariasis in this area even after controlling the location using stratified analysis. Therefore, the clustering of *A*. *lumbricoides* infection in schools close to the stream could be explained by higher temperature in communities located in the valley that accelerates the development of helminth eggs and predicts the prevalence of ascariasis [22]. Besides, the stream water may increase the moisture of the soil in its banks, providing a suitable environment for sustenance and development of Ascaris eggs [23].

Not washing hands and eating exposed food variables were independent risk factors of ascariasis, which are in line with findings reported from different areas in Yemen [15, 24–26], and other countries such as Saudi Arabia [27], Ethiopia [28], China [29] and India [30]. These findings necessitate providing school-based health education and implementing the MDA with albendazole together. Schoolchildren whose fathers are unemployed had higher infection rate of ascariasis, which was in agreement with previous studies conducted in Yemen [16, 31], Ethiopia [32], Ghana [33] and Nigeria [34]. This finding may be attributed to those unemployed fathers who most often work in agricultural fields with a probability of accompanying their children during the work, a common practice in the rural areas of Yemen, and exposing them to contaminated soil.

The prevalence of *S*. *mansoni* among schoolchildren in the present study was 6.5%, placing An-Nadirah District in the low-risk category [20]. However, the finding indicates no reduction in the prevalence of *S*. *mansoni* compared to the prevalence reported by latest remapping conducted in 2014 (5.4%) [15]. It is well known that treatment alone, the current strategy for prevention and control of schistosomiasis in Yemen, is not sufficient to achieve the interruption of the transmission and, therefore, environmental and educational interventions are recommended [20]. The unreduced prevalence *S*. *mansoni* in this community may be explained by environmental and human behavior–related factors such as the presence of a permanent water course in this district together with the water contact activities that favor the reinfection in spite of the regular preventive chemotherapy as observed elsewhere [35–37]. In this context, the study revealed that schoolchildren attending schools located close to the stream are at higher risk of *S*. *mansoni*, which was in agreement with previous studies conducted in Ethiopia [38], West Africa [39], and Sudan [40]. The permanent water stream, flowing in Bana Valley, might contribute to the high risk of infection by increasing presence and abundance of *Biomphalaria* snails shedding cercariae [41]. Therefore, reducing or eliminating intermediate snail hosts should be integrated as additional intervention for effective control strategy [42] beside preventing water contamination through improved sanitation [20, 43]. In addition, the focal nature of *S*. *mansoni* in this district suggests a reevaluation of subdistrict instead of district as the implementation unit for prevention and control of schistosomiasis. The focus on exposed communities at the district level will save resources particularly in Yemen, a country with limited resources and political instability.

Swimming was an independent factor associated with intestinal schistosomiasis among schoolchildren, which was consistent with previous studies conducted in Yemen [16, 24, 44] and other developing countries such as Egypt [45], Ethiopia [38, 46] and Sudan [40]. This finding could be attributed to the fact that swimming increases the duration of skin contact with water which causes skin penetration by *S*. *mansoni* cercariae. It is noteworthy that swimming is a recreation activity and WASH intervention may not have a significant impact on reducing swimming -related transmission. Thus, school-based health education should be a component of the control strategy of schistosomiasis [20, 47].

Beside abovementioned predictors that might contribute to unchanged prevalence of intestinal schistosomiasis, the impact of the ongoing war conflict on the regularity of the preventive chemotherapy should not be ignored. However, Ministry of Public Heath and Population conducted several preventive chemotherapy campaigns following the start of the civil unrest in 2015 with support from the World Bank [48] and the World Health Organization as mentioned in a conversation with A.A.Q Thabit (July, 2022).

## 5. Conclusion

Although preventive chemotherapy as a single intervention has achieved success in reducing the prevalence of *A*. *lumbricoides* by half in An Nadirah District, Ibb Governorate, there has been no reduction in the prevalence of *S*. *mansoni* since the latest remapping survey conducted in 2014. However, the district is still classified in the low-risk category. The permanent water stream flowing in the district valley together with swimming behavior of schoolchildren seem to play significant roles in sustaining the transmission of *S*. *mansoni*. Poor hygienic practices contribute to the occurrence of *A*. *lumbricoides*. For eliminating the transmission, school-based health education, snail control, school- and community–based WASH programme and mass drug administration should be components in the control strategy of schistosomiasis and STH. The focal transmission suggests focusing the interventions on exposed communities instead of targeting the whole district.
